# Muscle modifications in fibromyalgic patients revealed by surface electromyography (SEMG) analysis

**DOI:** 10.1186/1471-2474-10-36

**Published:** 2009-04-15

**Authors:** Laura Bazzichi, Marco Dini, Alessandra Rossi, Silvia Corbianco, Francesca De Feo, Camillo Giacomelli, Cristina Zirafa, Claudia Ferrari, Bruno Rossi, Stefano Bombardieri

**Affiliations:** 1 Department of Internal Medicine, Division of Rheumatology, S. Chiara Hospital, Pisa, Italy; 2 Department of Psychiatry, Neurobiology, Pharmacology and Biotechnology, University of Pisa, Pisa, Italy; 3 Department of Neuroscience, University of Pisa, Pisa, Italy

## Abstract

**Background:**

Several studies have been carried out in order to investigate surface electromyography (SEMG) response on fibromyalgic (FM) patients. Some studies failed to demonstrate differences between FM patients and healthy individuals while others found differences in SEMG parameters. Different muscular region have been analyzed in FM patients and heterogeneity is also produced because of the different ways in which the SEMG technique is used.

The aims of this study were to evaluate muscle modifications by SEMG analysis in FM women with respect to a sample of healthy controls and to investigate the relationships between SEMG parameters and the clinical aspects of the disease.

**Methods:**

SEMG was recorded in 100 FM women (48.10 ± 11.96 yr) and in 50 healthy women (48.60 ± 11.18 yr), from the tibialis anterior and the distal part of vastus medialis muscle during isometric contraction. Initial values and rate of change of median spectral frequency (MDF) and conduction velocity (CV) of the SEMG signal were calculated.

The clinical parameters "Fibromyalgia Impact Questionnaire", pain, tender points, tiredness were evaluated and the relationships between these data and the SEMG results were also studied. For the statistical analysis Mann-Whitney test, chi-square test and Spearman correlation were used.

**Results:**

MDF absolute values and the so-called Fatigue Index (FI) were significantly lower (p < 0.001) in both muscles studied in FM patients (MDF: 93.2 μV; FI: 1.10, 0.89) with respect to healthy controls (MDF: 138.2 μV; FI: 2.41, 1.66) and a smaller reduction in the percentage values of MDF was observed in FM patients vs controls (22% vs 38%). A significant correlation was found between the SEMG parameter decrement of normalized median frequency (MNF) (%) and seriousness of FM (evaluated by means of tender points).

**Conclusion:**

We have found some interesting muscle modifications in FM patients with respect to healthy controls, regarding MDF, CV and FI values which resulted significantly lower in FM. Patients might have a different fiber recruitment or a possible atrophy of type II fibers suggesting that they are not able to reach muscle relaxation.

## Background

Surface Electromyography (SEMG) is a non-invasive technique which can provide useful information regarding a muscle's functional status. The use of SEMG has become widespread because of its non-invasive characteristic (differently from classic EMG, it is needle free) and for the potentiality to explore muscle fibre type characteristics; in fact this method has been applied in the estimation of muscle fatigue phenomena, monitoring the physiological effects of rehabilitation and training [1-6].

Fibromyalgia (FM) is a chronic disorder characterized by widespread musculoskeletal pain and fatigue [7]. Patients with FM display low pain threshold levels at specific anatomic areas called tender points [8]. Most FM patients report disrupted or non-restorative sleep, mood disturbances, and several more syndromes (e.g., restless leg syndrome, irritable bowel syndrome and chronic headaches) [9,10], consequently physical and emotional health as well as quality of life is often seriously impaired [11,12].

For the peculiar characteristics of SEMG, it can be considered a useful technique in assessing the muscle status of FM patients and for analyzing some aspects of their peripheral fatigue. Conduction velocity (CV) and median frequency (MDF) are the two parameters measured by SEMG. Because they have been shown to be indirect measurements of the diameter of muscle fibers and indirect means for inferring the fiber type muscle constitution, SEMG may be used to assess the modifications of FM muscle.

A heterogeneous picture exists in literature about SEMG and EMG studies on FM patients, some studies failed to demonstrate differences between FM and healthy individuals [13-16], while others found differences in CV values and/or in other parameters [17-22]. Different muscular region have been analyzed in FM patients, furthermore heterogeneity is produced because of the different ways in which the SEMG technique is used.

In light of these results, we propose to assess the muscular response detected by SEMG at tibialis anterior and the distal part of vastus medialis muscle in a large cohort of FM patients, to compare the results with a sample of healthy controls, and to investigate the relationships between their muscle modifications and clinical aspects of the disease.

## Methods

### Subjects

100 consecutive women affected by primary fibromyalgia, aged 48.10 ± 11.96 yr (mean age ± S.D.), and 50 healthy women (48.60 ± 11.18 yr) were enrolled. Patients were recruited and clinically classified at the Division of Rheumatology, University of Pisa (S. Chiara Hospital) according to the 1990 American College of Rheumatology criteria (ACR criteria) [1], which include: pain for more than 3 months from all of the four body quadrants, axial skeletal pain and pain upon digital palpation of at least 11 out of 18 specific bilateral points. The healthy controls were either friends or neighbours of patients. Controls were allowed to report sporadic or situation-related minor headache and/or muscle aches because of the universal nature of such symptoms.

Exclusionary criteria for normal volunteers were: any of the above ACR criteria for fibromyalgia; use of any medication. Exclusionary criteria for patients were: the presence of a major clinical condition other than fibromyalgia. Were excluded from the study patients and controls with recent or past history of psychiatric disorders, neuromuscular pathology, metabolic and endocrinological disorders, kidney, hepatic and heart failure and pregnant females.

Patients and controls had comparable body mass index (BMI) and nobody of them was obese (data not shown). All the participants answered a questionnaire about physical activity, they resulted employed in half-time jobs or at home with small children for less than 4 h per day.

SEMG analyses were performed at the Unit of Neurorehabilitation, at the S. Chiara Hospital (Pisa).

Written consent was obtained from all patients and controls after a full explanation of the procedure.

### Evaluation of clinical parameters

Tenderness at tender points was evaluated in each subject using the Fischer dolorimeter [23]. A rheumatologist applied the instrument at a rate of 1 Kg/s and the patient was instructed to say when this procedure became painful. The pain threshold was calculated for 18 points, and the tender point (TP) count was determined by the number of tender points that had a threshold of ≤ 4 kg/cm^2^. The total fibromyalgic tender point score (right + left) was used in the statistical analysis.

Either patients or controls received a "Fibromyalgia Impact Questionnaire" (FIQ) containing 10 different items, each of them being evaluated to gave the FIQ total score [24]. Total score reflect the impact of fibromyalgia on quality of life, ranging from 0 (no impact) to 100 (maximum impact). For each patient an evaluation was also made of fatigue, stiffness and pain by means of a visual analogic scale (VAS).

### Myoelectric measurement and experimental procedure

The tibialis anterior and the distal part of vastus medialis muscle were selected for this study because of the relatively extensive body of data available on its structure and behaviour particularly suitable for conduction velocity (CV) measurements [25].

Patients and controls were examined lying on a bed with an articular angle of 135° for vastus medialis obliquus muscle and of 110° for tibialis anterior.

The force of the ankle dorsiflexion was recorded with an electronic strain gauge inserted in a specially foot isometric ergometer.

The motor point of the muscle were found by means of electrical stimulation and that point, with the lowest stimulation threshold providing the highest muscle contraction, visually registered, was chosen for the stimulation protocol. Stimulation consisted of pulse trains (each stimulus 0.3 msec in width) delivered at a frequency of 15 Hz and 35 Hz; the 15 Hz value is near the threshold for fused contraction while 35 Hz represents the highest frequency without M-waves overlapping.

A supramaximal stimulation 10–15% above the level generating the maximum amplitude motor-evoked potential was applied. The myoelectric signal was detected with the 4 bar electrode technique described by Broman et al. [26].

A single differential output was obtained from the 2 central bars and was used to compute the median spectral frequency (MDF). The detection technique provided 2 double-differential outputs from which the muscle fibre CV was estimated [26].

The electrode was moved over the muscle in the area between the most distal motor point and the tendon and then fixed with an elastic strap in a position that showed highly correlated double differential signals during the stimulations, according to Roy et al. [25].

When the detection electrode was applied correctly, and after a 5 min rest so that the subject did not feel any fatigue effect, stimulated contractions lasting 30 s were performed with the subject relaxed and physically passive; the absence of voluntary myoelectric signals (ME) signals indicated this state. For each subject two supramaximal isometric electrically elicited contractions, lasting 30 seconds, were evoked at the frequency of 15 Hz and 35 Hz, and a 10 min interval was allowed for recovery between contractions.

The stimulation artefact, which detrimentally affects the myoelectric signal, was suppressed electronically by means of a technique described by Knaflitz and Merletti [27].

CV and MDF rate of change (m/s^2 ^and Hz/s respectively) and their normalized values (%/s) were calculated.

The laboratory personnel were blinded as to the diagnosis (healthy control or FM) of the subjects, and the subjects were instructed not to disclose their diagnostic status. All the data processed before the statistical analysis was made without knowledge of the diagnostic status of the subjects.

### Data management

The Median Spectral Frequency (MDF, Hz) and Conduction Velocity (CV, m/s) were computed off-line with numerical algorithms according to the methods described by McGill and Dorfman [28] and Merletti et al. [29], respectively, then tabulated and plotted versus time for each stimulated contraction.

The MDF and the CV data values collected from FM subjects and controls showed a curvilinear trend over time, which could be modelled with a polynomial curve of type y = at^2 ^+ bt + c.

A normalized fatigue index (FI) as 100b/c, was defined, where: b is the value of the slope of the polynomial curve for t = 0, and c is the initial value [30].

### Statistical analysis

A polynomial curve was applied to the data to calculate the initial value and rate of change of MDF and CV. The polynomial curve model was shown to fit the experimental data better than the exponential model. The normalized rate of change for each variable was calculated as the percentage ratio between rate of change and initial value.

For the statistical analysis of the data, Mann-Whitney test, chi-square test and Spearman correlation were used. Two-tailed p values less than 0.05 were considered to be significant.

## Results

Background variables of FM patients and controls are shown in Table 1.

**Table 1:** Background variables of FM patients (N = 100) and controls (N = 50). (Values are expressed as mean ± SD).

	**FM**	**Controls**
Age (years)	48.10 ± 11.96	48.60 ± 11.18

Duration of disease (years)	6.04 ± 6.12	-

FIQ	60.95 ± 17.48	8.50 ± 5.72

TP	15.49 ± 4.33	2.75 ± 1.80

Fatigue	7.15 ± 2.46	0.25 ± 0.40

Pain	7.01 ± 2.46	0

Stiffness	6.95 ± 2.45	1.60 ± 2.10

FIQ: Fibromyalgia Impact Questionnaire; TP: tender point.

Regarding SEMG results only the results of the frequency of 35 Hz are shown. In Tab. 2 are shown the mean ± SD of MDF, CV and FI values calculated on all patients and controls at time 0 (t = 0, basal) and after 30 secs (t = 30); there are significant differences between the mean MDF absolute values at basal (time = 0) (93.2 ± 6.0 μV vs 138.2 ± 5.1 μV) and the so-called Fatigue Index (FI, 1.10 vs 2.41; 0.89 vs 1.66), which all resulted significantly lower (P < 0.0001) in fibromyalgic patients with respect to controls.

**Table 2:** Absolute basal (t = 0 secs) and final values (t = 30 secs) (mean ± SD) of MDF, CV and FI of FM patients and controls.

	MDF (μV) FM	CV (m/sec) FM	MDF (μV) controls	CV (m/sec) controls
Basal (t = 0 secs)	93.2* ± 6.0	4.0 ± 0.5	138.2* ± 5.1	5.1 ± 0.5

Final (t = 30 secs)	72.6 ± 3.9	3.1 ± 0.3	86.1 ± 2	3.4 ± 0.3

Fatigue Index	1.10*	0.89*	2.41*	1.66*

MDF: median spectral frequency; CV: conduction velocity

MDF and CV are expressed as absolute values. Mean ± SD of MDF, CV and FI are calculated

on 100 patients and 50 controls.

*Basal MDF controls vs basal MDF FM, p < 0.0001

*Fatigue Index controls (MDF and CV) vs Fatigue Index FM (MDF and CV), p < 0.0001

**Figure 1:**
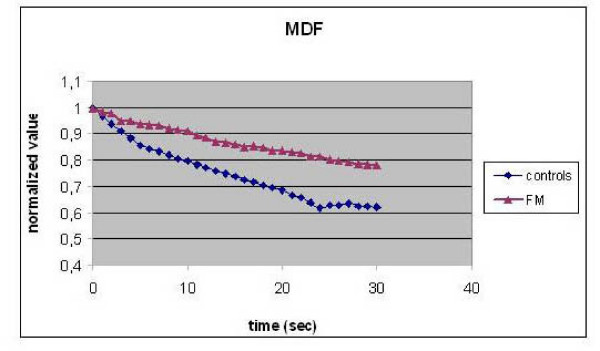
**Normalized values of MDF in FM patients and controls (0–30 sec)**.

**Figure 2:**
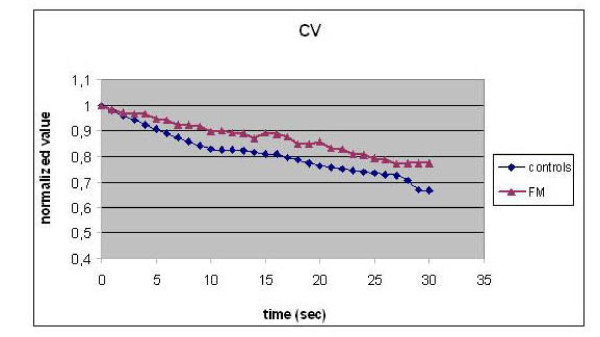
**Normalized values of CV in FM patients and controls (0–30 sec)**.

Figs. 1 and 2 show the typical response of SEMG (MDF and CV respectively, normalized values) in 100 FM patients and in 50 controls, at the frequency of 35 Hz.

A significant smaller reduction (p < 0.001) of MDF (%) and CV (%) was found in FM patients with respect to controls.

The response of SEMG at the frequency of 15 Hz is not shown because there was no statistically significant difference between FM patients and the control group at this stimulation frequency.

A significant and negative correlation was found in FM patients between the % of MDF decrement and the number of TPs (r = -0.44, P = 0.0001). Non-significant correlations were found between the SEMG parameters FI, %MDF, %CV and age, duration of disease, FIQ, fatigue, pain and stiffness.

## Discussion

In our study a different muscular response detected by means of SEMG at the tibialis anterior and the distal part of vastus medialis muscle has been demonstrated in patients affected by fibromyalgia. FM patients exhibited lower MDF and CV absolute values with respect to healthy controls and the main difference revealed involves the rate of CV and MDF change during stimulated contraction, which was lower in FM patients. A smaller reduction in their percentage values was observed in FM patients with respect to controls (22% vs 38%). In particular, FM patients sustained an apparent smaller fatigue related decrease in both MDF and CV values compared to controls, which let us suppose muscle modifications in FM patients. Also Fatigue Index values resulted significantly lower in FM.

In normal subjects the CV values decrease during prolonged contraction and this is an electrical manifestation of fatigue [31]. The decrease, is proportional to the ratio of type I/type II fibres; that is, the greater the number of type II fibres, the greater the decrement. This provides us with information about the fibre-type distribution. MDF is influenced by muscle fibre diameter and fatigue phenomena and is generally accepted as one of the power spectrum parameters most suitable for tracking spectral compression due to localized muscle fatigue [32] and MDF modifications during stimulated contraction are strictly related to reductions in CV [31]. In the light of the above mentioned notions, we may suppose the existence of muscle modifications in fibromyalgic patients, in particular of type II fibers, the white rapidly contracting type fibers.

Several studies that investigated SEMG activity failed to demonstrate differences between FM patients and healthy controls [13-16,33]. Other papers reported a difference between values of CV and/or in other parameters of SEMG activity during isometric exercise in patients with FM and healthy controls [17-22]. Furthermore in literature is present a great heterogeneity about the SEMG technique applied, however our results are in accordance with earlier findings that patients with chronic pain have increased muscle tension.

The parameters % MDF decrement correlated significantly and negatively with the number of TPs, linking a clinical index with an instrumental measure of muscle modification. That is, when patients feel worse, we may suppose a smaller recruitment or an atrophy of type II fibres.

In a previous research, one of us examined the muscle changes occurring in Parkinson's disease (PD) [34] and found that the main difference observed between PD subjects and controls was the rate of change of MDF and CV during the course of stimulated contraction; patients with PD, like FM patients, sustained a smaller fatigue related decrease in both parameters compared to controls. Furthermore muscle biopsy of patients with PD was performed in this study and histological data showed a tendency towards hypertrophy of red type I fibres, rich in oxidative enzymes, and atrophy of type II fibres. The muscle changes occurring in Parkinson's disease have been hypothesized as a consequence of the modified pattern of motor unit activation and rigidity, which are characteristic of the disease. FM and PD disease are different in many aspects, and it is not easy to explain, but our SEMG results showed similar muscular response, which let us suppose a similar metabolic state.

In the late 1980s several studies regarding muscle biopsies of FM patients were carried out. Atrophy of type II fibres has been reported in several studies and in different muscular regions [35-37]; studies of the normal trapezius muscle indicate a relatively poor supply of capillaries as well as low mitochondrial volume density compared to limb muscles [38]. From this point of view we may hypothesize that the problems of FM patients might be due to a consequence of the modified pattern of motor unit innervation. FM patients show an activation pattern similar to PD patients, with a possible atrophy of type II fibres. In this context, paradoxically, anaerobic exercise would seem useful in muscular rehabilitation of FM patients.

In accordance with this hypothesis, recent studies support the hypothesis of a dysfunction of dopaminergic neurotransmission in fibromyalgic patients [39] and indicate that it represents an important relevant target for the treatment of fibromyalgia. Dopamine is best known for its role in pleasure, motivation and motor control and in the basal ganglia it may also be important for pain modulation, where it can be released in response to noxious stimulation and leads to endogenous antinociception. Dopaminergic agonists of receptors D3/2 have been efficacious in decreasing the symptoms of fibromyalgia; Holman [40], Holman and Myers [41], in a pilot double blinded study, reported a decrease of pain evaluated by visual analogic scale (VAS) and improvement in other parameters such as rigidity and myalgic score of TPs after the treatment with ropinirole and pramipexole.

## Conclusion

A heterogeneous literature exists about the matter if chronic pain conditions are associated with changes in CV, firing rate, measured by SEMG. In the present study of severe chronic pain condition like fibromyalgia and healthy controls, we have found some interesting muscle modifications in FM patients with respect to healthy controls, regarding MDF, CV and FI values which resulted significantly lower in FM. In particular it seems that FM patients show an activation pattern of motor unit similar to that of patients affected by PD disease, with a possible atrophy of type II fibers.

SEMG, which is a non invasive technique and needle free, from this point of view might be useful in assessing the modifications of muscle characteristics in FM patients and it might be an instrument to support diagnosis, to individuate target therapies and to evaluate disease evolution and improvement during a period of time.

## Competing interests

The authors declare that they have no competing interests.

## Authors' contributions

LB and AR have been involved in drafting the manuscript and revising it critically for important intellectual content. LB, BR and SB have given final approval of the version to be published. MD and SC have made substantial contributions to acquisition of data. FDF and CG participated in the design of the study and performed the statistical analysis. CZ and CF have made substantial contributions to analysis and interpretation of data. All authors read and approved the final manuscript.

## Pre-publication history

The pre-publication history for this paper can be accessed here:


